# Novel Morphological Glial Alterations in the Spectrum of Prion Disease Types: A Focus on Common Findings

**DOI:** 10.3390/pathogens10050596

**Published:** 2021-05-13

**Authors:** Moisés Garcés, Isabel M. Guijarro, Diane L. Ritchie, Juan J. Badiola, Marta Monzón

**Affiliations:** 1 Research Centre for Encephalopathies and Transmissible Emerging Diseases, Institute for Health Research Aragón (IIS), University of Zaragoza, 50013 Zaragoza, Spain; moisesgarces1@gmail.com (M.G.); isabelmariagt91@gmail.com (I.M.G.); badiola@unizar.es (J.J.B.); 2 National CJD Research & Surveillance Unit, Centre for Clinical Brain Sciences, The University of Edinburgh, Edinburgh EH4 2XU, UK; diane.ritchie@ed.ac.uk

**Keywords:** astroglia, microglia, interlaminar astroglia, prion types, cortex, cerebellum

## Abstract

Human prion diseases are a group of rare fatal neurodegenerative diseases with sporadic, genetic, and acquired forms. They are neuropathologically characterized by pathological prion protein accumulation, neuronal death, and vacuolation. Classical immunological response has long been known not to play a major in prion diseases; however, gliosis is known to be a common feature although variable in extent and poorly described. In this investigation, astrogliosis and activated microglia in two brain regions were assessed and compared with non-neurologically affected patients in a representative sample across the spectrum of Creutzfeldt–Jakob disease (CJD) forms and subtypes in order to analyze the influence of prion strain on pathological processes. In this report, we choose to focus on features common to all CJD types rather than the diversity among them. Novel pathological changes in both glial cell types were found to be shared by all CJD types. Microglial activation correlated to astrogliosis. Spongiosis, but not pathological prion protein deposition, correlated to both astrogliosis and microgliosis. At the ultrastructural level, astrocytic glial filaments correlated with pathological changes associated with prion disease. These observations confirm that neuroglia play a prominent role in the neurodegenerative process of prion diseases, regardless of the causative prion type.

## 1. Introduction

Creutzfeldt–Jakob disease (CJD) is a group of progressive neurodegenerative pathologies characterized by long incubation periods and an inevitable fatal outcome. The causative agent is generally held to be the accumulation of misfolded prion protein (PrP^sc^) [1]. PrP^sc^ deposition in the brain is a hallmark, but vacuolation, neuronal loss, and gliosis are all prominent neuropathological features. Although the lack of immune response was classically associated with prion disease pathogenesis, glial involvement has been consistently seen [2,3,4,5,6,7,8] leading to speculation that glia are crucial players in prion diseases in accordance with the general neuroinflammatory hypothesis [9,10,11,12]. 

Some authors tend to focus on microglia considering them responsible for the neurodegenerative process characteristic of this group of diseases, in part because microglia are the main immune cell of the central nervous system (CNS) [13,14,15]. Their pleomorphism is widely accepted. Studies of their morphological alterations have suggested a continuum of changes, from resting to an activated state or as consequence of neuropathological states, including different morphologies: Ramified, activated, amoeboid, dystrophic, and rod-like in neurodegenerative disease [16,17,18]. Evidence indicates that this activated cellular type, besides contributing to the clearance of damaged cellular material and protein aggregates, might induce a neuroinflammatory state, which accelerates the development of neurodegeneration [19]. Nevertheless, a definitive view on the role of the activation state of these glial cells in the neurodegeneration process remains elusive. 

In contrast, astroglia were initially considered as building a cellular network in order to support neurons [20]. However, some studies established close interaction between these glial cells, microglia, the and brain blood barrier (BBB) [21,22] as well as their involvement in essential functions for CNS maintenance [20,23]. Additionally, some other studies provided additional evidence that astrocytes might contribute to the pathogenesis of neurodegenerative diseases [24,25,26,27] have been also published. 

Based on this lead role of gliosis as the key regulator of neuroinflammation, some studies so far have addressed the issue of astrocytic and microglial activation in prion diseases, at the same time establishing a potential neurotoxic [14,15,24,28] or neuroprotective role [7,29,30] for both cellular types.

On the other hand, the widely known phenotypic heterogeneity of prion diseases establishes the existence of several CJD types depending on origin (genetic, acquired, or sporadic) [31]. This variability depends in part on the existence of individual prion strains. Prion strains are defined as natural infectious isolates characterized by distinctive clinical and neuropathological features that are faithfully recapitulated in animal models by transmission studies [32]. The host genotype, in particular the (Met/Val) polymorphism at codon 129 of the human prion protein gene PRNP along with the PrPres isoform, is an also important factor that influences sCJD heterogeneity [33]. Efforts have been made to establish glial profiles to contribute to prion strain characterization [7,34,35,36]. Franceschini et al. [37] have recently proposed microgliosis profiling as a valid component of molecular and histopathological typing. In contrast, our recent study focused on the preliminary analysis of the astroglial and microglial activation profiles of which brains from sCJD and fCJD presented some profiles in common [38]. To our knowledge, an exhaustive study of the possible relationship among glial profiles and findings constituting neuropathological hallmarks of prion diseases encompassing cases of different CJD types have not been reported. Although morphological studies are considered to only provide descriptive results by some authors, a link between neuroinflammation and glial phenotypic changes in neurological diseases constitutes a major current area of research interest.

Therefore, in order to contribute to a clearer understanding of the actual role of host immune response in prion diseases, we have chosen to characterize the morphological, distribution, and intensity profiles of astroglial and reactive microglial populations as agents of the neuroinflammatory process in the different human prion diseases (genetic, gCJD; iatrogenic, iCJD; variant, vCJD; sporadic, sCJD; and variably protease-sensitive prionopathy, VPSPr). Each profile is correlated with histopathological findings and PrP^sc^ accumulation in cerebellum and frontal lobe samples as one of the most and the least affected encephalic areas, respectively. In contrast to previous studies, our aim consisted of paying particular attention to features common to all CJD types regarding pathological changes instead of diversity among them. 

## 2. Results

### 2.1. Histopathological Findings (Spongiosis and PrP^sc^ Accumulation) and Glial Activation

**Figure 1:**
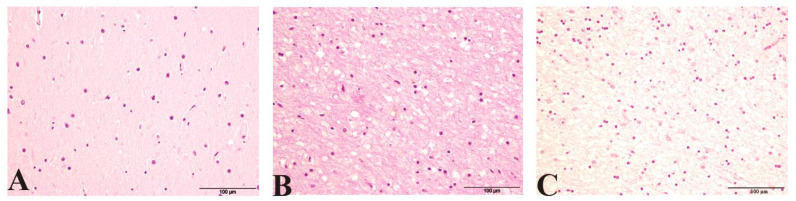
Hematoxylin eosin staining (H/E) showing (**A**) No evidence of spongiosis in a control. (**B**) Typical spongiform change associated with CJD. (**C**) Spongiosis status compatible with panencephalopathic CJD in iCJD MM1.

**Figure 2:**
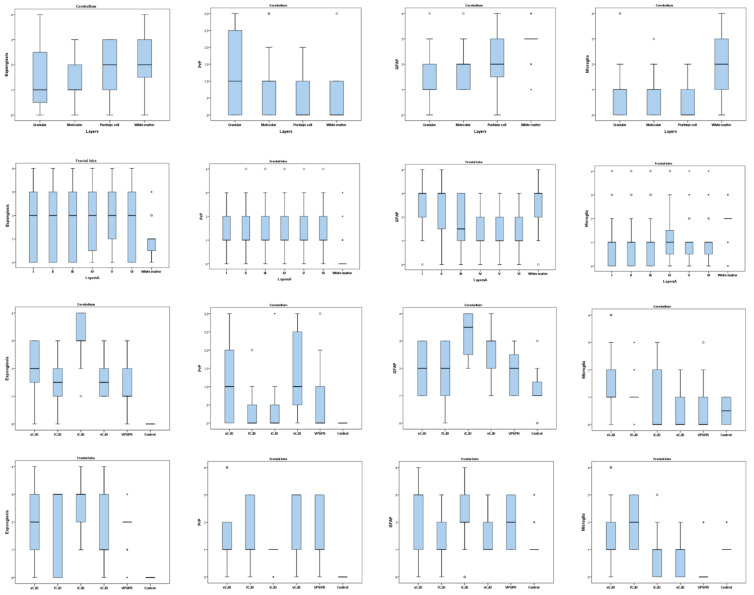
Comparison of spongiosis, PrP^sc^ deposit, astrogliosis, and reactive microglia scores from the cerebellum and frontal cortex in different layers and CJD types. Box plots represent median (Me) and interquartile range (IQR); * *p* < 0.05.

Spongiform changes were seen in all CJD samples by H/E staining but were absent in the three control cases (Figure 1). The severity of spongiform change was highly variable among cases reaching the highest severity in iCJD regardless of the brain region examined (3.08 ± 0.90 in cerebellum and 2.57 ± 0.93 in frontal cortex; Figure 2). One iCJD MM1 case showed a spongiosis status compatible with panencephalopathic cases of sCJD.

**Figure 3:**
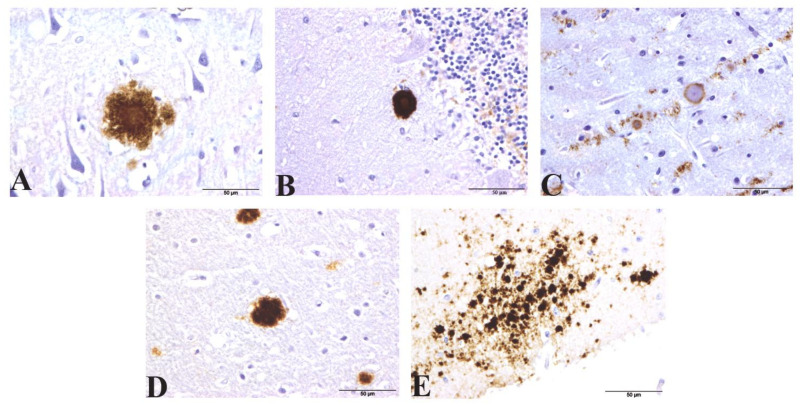
Protein aggregates detected by IHC for PrP^sc^ (12F10 antibody) in samples from different CJD type affected patients. (**A**) Kuru-like plaque in the frontal cortex from iCJD (hGH). (**B**) Kuru plaque in molecular layer from sCJD. (**C**) Unicentric plaque with a PrP^sc^ halo atypically surrounding a core in vCJD. (**D**) Kuru-like plaque in iCJD (hGH). (**E**) Microplaques in the molecular layer of cerebellum of VPSPr.

As expected, white matter showed a lower PrP^sc^ accumulation in comparison to grey matter in both brain regions in all cases examined (0.46 ± 0.72 in cerebellum and 0.33 ± 0.76 in frontal cortex). Consistent with previous reports, a wide variety of different PrP^sc^ deposits were found, with coalescent and granular patterns of accumulation predominating in both brain regions. In the frontal cortex, perivascular and pericellular deposits consistent with glial cell staining was found in 2/3 vCJD samples. The patterns of PrP^sc^ observed in the different sCJD subtypes was constant to that published previously. For example, perivacuolar PrP^sc^ deposits were detected in their frontal cortex, although also to a lesser degree in two vCJD samples. Additionally, different kinds of protein aggregates were also highlighted: Kuru plaques were detected in the granular and molecular layer of all three sCJD MV2 cases and florid plaques in frontal cortex/cerebellum of all three vCJD cases. Kuru-like plaques were also detected in cerebellum and frontal cortex samples from iCJD (GMT, MV1) and iCJD (GHT, MV1+2), respectively. Atypical unicentric plaque-like accumulations were seen in vCJD and also multicentric ones in some sCJD cases. Microplaques within the molecular layer of the cerebellum were detected in one sample of VPSPr (Figure 3). PrP^sc^ was not detected in any control samples.

Glial activation significantly increased, reaching the highest values of astrogliosis (2.96 ± 0.69 and 2.54 ± 0.93) as well as microgliosis (1.92 ± 1.02 and 1.92 ± 0.78) in the cerebellum and frontal cortex, respectively. Surprisingly, when microglial activation was assessed with respect to different types, no statistical differences were found. However, they were found when astrogliosis was studied (*p* < 0.001). 

### 2.2. Evaluation of Glial Profiles in Different CJD Types

Overall, the degrees of GFAP and activated microglial marker immunoreactivity correlated with the individual CJD types in both regions of the brain (*p* < 0.001 in all cases except for microglia in cerebellum where *p* = 0.007; Figure 2). The highest GFAP immunostaining was found in iCJD (3.25 ± 0.87) and sCJD (2.29 ± 1.02) cerebellar and cortical samples, respectively. Microglial immunoreactivity showed low intensity in all samples but was especially low in iCJD, vCJD, and VPSPr where they ranged 0.50–0.83 in cerebellum and 0.29–0.76 in frontal cortex. The highest values were found in cerebellum from sCJD (1.47 ± 1.21) and frontal cortex from fCJD (1.95 ± 0.80).

### 2.3. Correlative Analysis between Glial Activation and Neuropathological Lesions

Interestingly, PrP^sc^ deposition was not related to any glial profiles (Table 1). However, spongiosis correlated to the activation of both astroglial (R = 0.164; *p* = 0.008) and also reactive microglial (R = 0.205; *p* = 0.001) populations. Moreover, the activation of both of these glial cells highly correlated with each other (R = 0.219; *p* = 0.000).

#### 2.3.1. Glial Morphological Changes

**Figure 4:**
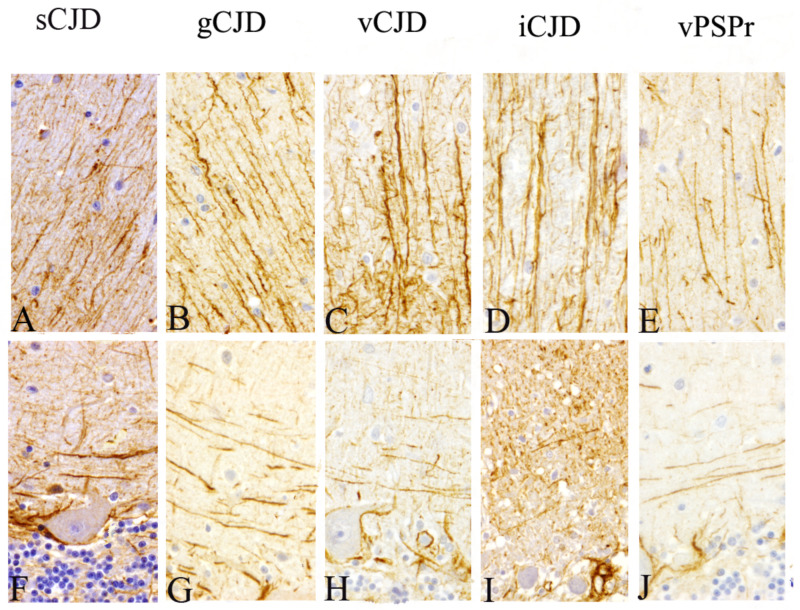
Immunohistochemical patterns observed when astroglial marker (GFAP antibody) was assessed in sagittal cerebella corresponding to each CJD subtype. Alternative (**A**–**E**) radial and (**F**–**J**) horizontal (parallel to the pial surface) profiles were consistently observed in the molecular layer (Magnification: 400×).

Similar to that found in our previous studies [38,39,40], all pathological cerebella shared two alternative GFAP immunostaining profiles in molecular layer. These consisted of a mainly radial pattern when astroglial intensity was higher or matching varicose fibers parallel to the pial surface when it was lower. As a novelty, these profiles were observed irrespective of strain (Figure 4). 

**Figure 5:**
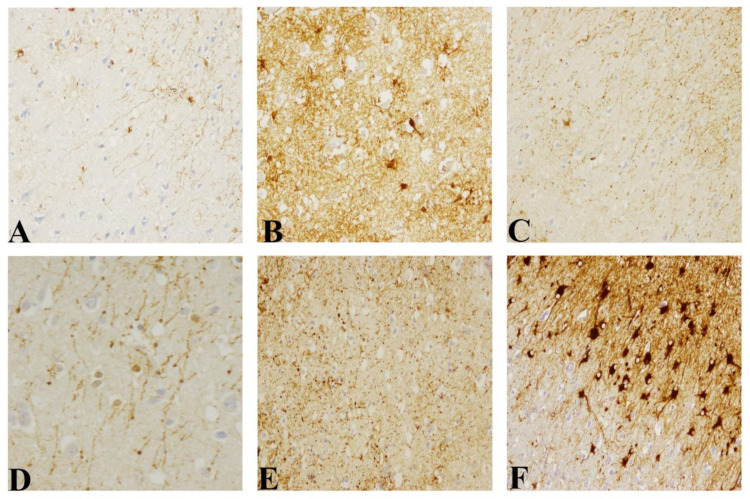
In comparison with that in healthy control, (**A**) morphological changes of interlaminar astroglia in CJD cases are illustrated (GFAP antibody). (**B**) Replacement by typical stellate astroglial cells with increase of astrogliosis. (**C**) Increase of number of terminal masses of interlaminar astroglia. (**D**) Increase of diameter of terminal masses. (**E**) Disruption in I–III frontal cortex. (**F**) Gemistocytic phenotype in panencephalopathic CJD (Magnification: 200×, except for (**D**) 400×).

Concerning the frontal cortex in control samples, mild GFAP immunostaining in the meninge and in the subpial area mostly coinciding with the supragranular layers (I, II, and III layer) were found. Additionally, interlaminar astroglia appearing as palisade fibers with radial morphology were easily identifiable covering I to IV layers. In samples from CJD affected patients, an increase of GFAP immunostaining in the meninge as well as the number of interlaminar astrocytic bodies in the I layer and of astrocytes surrounding blood vessels in deeper layers were found. The most outstanding alterations were focused on interlaminar glia (Figure 5). These peculiar glia showed several morphological changes that varied from disorganization or fragmented process to disappearance and/or replacement by reactive stellate astrocytes, increase of number of terminal masses or disruption. In the panencephalopathic case, the highest reactive morphology, known as gemistocytic astrocytes with a spherical pale nucleus, and cellular extensions were identified. The lack of cytoplasmic prolongation and hyper reactivity was highly marked in white matter. Additionally, in that case, the interlaminar astroglia showed the thickest process despite the destruction of typical cytoarchitecture of the frontal cortex.

**Figure 6:**
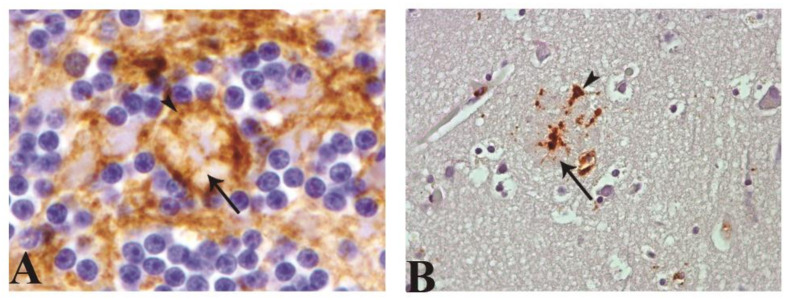
Illustration of glial cells associated to protein deposits by astroglial (GFAP antibody; arrowhead) and microglial (CD68 and MHCII antibodies; arrowhead) immunostaining, respectively. (**A**) Astrocytic process associated with Kuru plaque (arrow) in granular layer of cerebellum. (**B**) Microglial process associated to protein aggregation or clustered morphology associated with protein deposit (arrow; magnification: 40×).

Astroglial immunostaining always appeared associated with several protein aggregates, not exclusively kuru plaques. Meanwhile, microglial labeling was found to be associated with protein aggregation or clustered morphology associated with protein deposit, but only in scarce occasions (Figure 6).

**Figure 7:**
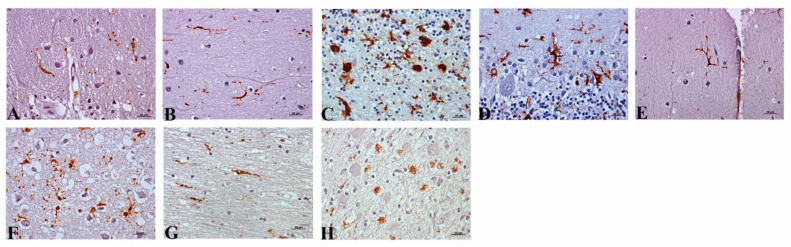
Reactive microglial morphology found in CJD cerebella (CD68 and MHCII antibodies). (**A**) Dystrophic. (**B**) Rod-like. (**C**) Amoeboid. (**D**) Hypertrophic (**E**) Ramified. Furthermore, those also found in the frontal cortex (**F**) Dystrophic. (**G**) Rod-like. (**H**) Amoeboid.

Ramified reactive microglia scattered across all of the examined areas in control cerebella was found, except for those cerebella that had granular layer degeneration in which amoeboid appearance was evidenced. In CJD cases, different morphological patterns were also associated with intensity degree in this brain region: Amoeboid, activated ramified, rod-like, and dystrophic (Figure 7). Microglia in the Purkinje cell layer remained in a ramified pattern unlike those in white matter, frequently showing amoeboid shapes. All healthy control cortex samples showed a sparsely scattered microglial immunostaining with a ramified morphology (except for some cases where it was dystrophic). In contrast, in all CJD samples from this encephalic area, a dystrophic pattern was consistently found. Amoeboid, rod-like and clustered (in association with protein deposits), were only present in some rare examples.

#### 2.3.2. Ultrastructural Findings

Samples showed well-preserved membranes, mitochondria crests, and homogeneous content in the matrix; however, because they came from tissue banks, they showed a much poorer preservation of their ultrastructure in comparison with cases that are fixed with aldehydes. Moreover, it is well recognized that the spongiotic changes associated with the CJD neurodegenerative process result in tissue damage of the sort seen in the micrographs shown here. This makes it harder to distinguish pathologic lesions from fixation artifacts at the sub-cellular level.

**Figure 8:**
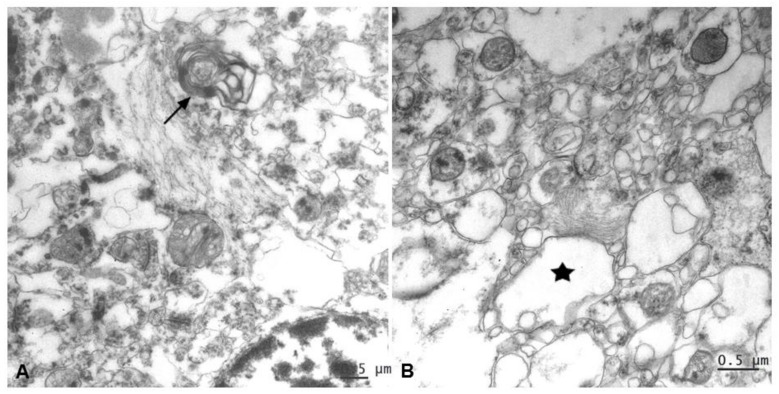
An evident increase of astroglial filaments was found to be associated with two essential neuropathological lesions in prion diseases. (**A**) Alteration of myelin (arrow) and (**B**) vacuolation (*, membrane disruption and curled fragments inside).

Nevertheless, all CJD samples allowed observing distinctive ultrastructural pathological changes of prion disease in relation with intermediate gliofilaments. This cytoskeletal structural filament, as the most characteristic component in astrocytes, was found located in close association with lesions such as vacuolation and alterations of myelin (Figure 8). 

## 3. Discussion

The hallmark neuropathological features of prion diseases are spongiosis, neuronal loss, misfolding prion protein deposits, and gliosis. Traditionally, pathological prion protein accumulation and its relation to neuronal death have dominated prion research, despite gliosis being a prominent feature in prion-affected brains. Consequently, the immunological function of glial cells had been a neglected aspect of research in this group of diseases.

Nevertheless, in recent years, the neuroinflammatory hypothesis has suggested that the host immune system is profoundly involved in neurodegeneration, playing an accelerating or even causative role in this process [9,12,41]. Inflammation implicates the modulation of immunological molecules (cytokines and interleukins), blood brain barrier (BBB) breakdown, and leukocyte infiltration. This last characteristic is not found in prion diseases nor other neurodegenerative diseases and constitutes a major difference from the classic inflammation found in other brain pathologies, such as those caused by infectious pathogens. 

The present study focused on reactive astroglia and microglia as the major glial cells involved in the immune response and, consequently, in the process of neuroinflammation. We chose to examine the cerebellum, as one of the most affected encephalic areas, and the frontal cortex, as a brain region where prion propagation is sometimes less pronounced in certain CJD types. Furthermore, it is relevant to point out that biopsy and autopsy samples from both of these brain regions are commonly used in the diagnosis of clinical CJD cases. An exhaustive study has recently been published seeking to establish whether different glial profiles support molecular and histopathological typing for prion strain characterization [37]. In contrast, the study presented here represents an attempt to establish, for the first time, what specific variations of neuroglia activation form a common denominator found for all prion types. 

This and other previous published quantitative studies have demonstrated a pronounced heterogeneity of glial distribution and intensity among different CJD types, probably in accordance with the individual host immune system participation. Perhaps it is also because, as known, neuroinflammation is not homogeneous, affecting cerebral areas differentially, and that is why glial cells, although being defined as a fundamental part of the process, do not behave uniformly in all cases. If a lineal process is involved, then case-to-case and regional differences may represent different stages in the neuroinflammation process. Several actors would take part in this event of neuroinflammation. The close correlation between astrogliosis and microgliosis demonstrated in this study would support this hypothesis, indicating that a combination of both glial types underlie neuroinflammation by mediating, exacerbating or failing to prevent the toxic effects of protein accumulation in the brain. Additionally, the close correlation of glial hyperplasia/hypertrophy and spongiosis in prion disease, as confirmed by ultrastructural examination in this and our previous studies on animal [42,43] as well as human prion [38] affected brains, strongly supports the essential role of neuroglia in the neurodegenerative process. Taken together, it would support a potential cooperative action between astrocytes and microglia in the final neuronal damage, as previously suggested by some other authors [27]. 

The microglial role in prion disease remains controversial. It has been established previously that there is a strong relationship between widespread deposition of PrP^sc^ and microgliosis in different experimental models [5,13,44,45]. However, in spite of the association between microgliosis and PrP^sc^ deposits in sCJD subtypes, some exceptions are also found, such as the MMT2 subtype [37]. Moreover, specific brain regions where PrP^sc^ has been detected by highly sensitive assays such as RT-QuIC did not show reactive microglia [46], supporting our statistical results questioning the association between microgliosis and widespread PrP^sc^ deposition depending on CJD types. Although at first glance the results presented here might appear conflicting, since microglia intensity and extension were assessed layer by layer in two specific brain areas (cerebellum and frontal cortex), we also focused on the whole CJD spectrum (genetic, sporadic, and acquired), and these studies provide reliable and novel complementary conclusions for contributing to the knowledge of the real microglial lead in CJD progression.

This lack of correlation between PrP^sc^ deposits and microglial profiles is consistent with the idea that not only misfolded protein deposit causes neurodegeneration. It is in conflict with the hypothesis that PrP^sc^ alone is toxic to neurons, without any involvement requirement of these historically supporting cells. Some authors also found no association between spongiosis and PrP^sc^ deposition in CJD tissues [47,48]. This along with the fact that gliosis is related to vacuolation confirms that other pathological events could occur in prion disease. A much more complex series of process may be involved including glial cells as main characters in the neuroinflammatory process [15]; or perhaps playing a neuroprotective role at least during the early stages [29,49]. 

Previous morphological studies of astroglial changes [38,39] constituted a useful tool for investigating prion pathogenesis by seeking evidence of common profiles shared by all studied CJD cases. The present study confirms that there are common features regardless of prion types. Several studies had been focused on the involvement of this cellular type in prion diseases by using cell cultures or experimental animal models; however, their real role is still unknown perhaps in part because of scarce studies focused on how they act in vivo [50]. Astrogliosis, known as hypertrophy and/or hyperplasia of astrocytes, is commonly observed along with the increase of neuropathological findings, although unaffected brain areas occasionally show high astrogliosis too [4]. It is known that astrocytes are the first cells that show PrP^sc^ deposits in prion disease progression [2], with it being possible to infect mice whose own PrP^c^ is exclusively expressed in astrocytes, besides research studies pointing out close relationship between infection susceptibility and PRNP genotype of iPSC-derived astrocytes [8]. Only few of them deal with the different CJD types and none in relation to the glial alterations common for all of them. 

In biology, a key idea is that structure determines function. Therefore, a profound alteration of morphology in glial cells implies major changes to cellular function and physiology [51,52]. In Alzheimer´s disease, astrogliosis seems to refer to morphological glial changes instead of cell proliferation [53]. These, along with our findings described here, support the significance of this contribution to the knowledge about glial changes in order to approach pathogenesis of neurodegenerative diseases.

It is widely known that the pleomorphic properties of microglial cells, as well as the presence of typical microglia morphologies, are associated with health and disease. The traditional term ‘resting’ microglia (i.e., ‘not activated’ microglia) may be misleading [18] because these cells elaborate many fine processes forming glial networks that regulate physiological events [54]. Referring to amoeboid or hypertrophic microglia, these reflect activation that indicates functional changes in response to pathological conditions [16] Some other phenotypic changes such as dystrophic changes could affect tissue homeostasis. Currently, the precise meaning of each of all these phenotypic changes constitutes a real challenge to neuropathology [18]. 

Specifically, in this study about prion diseases, a total of five morphologies have been reported. First, amoeboid morphology as a macrophage-like morphology related to enhancing activation and phagocytosis [17]; ramified, characterized by many fine processes, in spite of being named ‘resting microglia’ because it is present in healthy state, was also present in pathological events; and hypertrophic, showing thickened soma and shortened cellular prolongations, which could represent an intermediate state of ramified microglia in activation gradient [55]. 

Morphology known as rod cells or rod-like has been also detected in this study as markedly elongated, with scanty cytoplasm, and in a few processes [17]. Their function is largely unknown, but it is speculated that it could indicate a protective role [56,57]. Typically associated with chronic diseases such as neurosyphilis, subacute sclerosing panencephalitis, brain injury, and neurological diseases [55,57], rod cells are also present in neurodegenerative disease and aging [55]. As described here, this could be explained as senescent morphology since most patients develop CJD in old age. However, this question still remains unclear.

The last morphology found here, dystrophic morphology, characteristic of aging, is recognized as senescent and is occasionally detected in AD cases (with mild neuroinflammation). This is the first time it has been reported in prion diseases. Although the concept of ‘diseased microglia’ is raised in CJD [18,58], this kind of microglia was characterized as having an atypical morphology, showing intracytoplasmic vacuolation, spheroid fragmented cytoplasmic processes, and loss of distal branches. It has also been associated with cellular malfunction, lost homeostasis surveillance, and final neurodegeneration [59,60]. The frequent presence of this dystrophic microglial morphology would suggest a potential neurotoxic role for this cell in a neuroinflammatory context, in contrast to a neuroprotective role. This could be due to: (1) Microglial cells potentially reacting to specific misfolded protein (PrP^sc^) and inducing morphological changes along with hyperplasia cellular that finally imply detrimental effect, or (2) the presence of this dystrophic microglia could involve a loss of tissue homeostasis in addition to cellular function. 

On the whole, all these morphological changes could represent a continuum pathological event between minimally to maximally activated microglia, including many gradations [16,18].

Regarding astroglial morphology, the morphological change between radial and varicose in the molecular layer of cerebellum had been reported in our previous studies regarding the natural Scrapie model [61], gCJD and sCJD [38], and prion-like diseases [39,40]. Nevertheless, these results have been observed here for first time irrespective of prion type, as it was found in all CJD subtypes, including VPSPr.

Perhaps the most outstanding finding highlighted here is those morphological changes in interlaminar astroglia detected in the frontal lobe for first time in all CJD types. This cellular type is an astroglial population exclusively located in the frontal cortex of non-human primates [62] and humans [63] whose role remains unknown. In the healthy state, it forms palisade or cable-like structures that cover layers one to four of the cerebral cortex. Neurological diseases such as Alzheimer’s disease [64], Down syndrome [65], and psychological disorders such as bipolar disorder [66] have been reported to show alterations in this peculiar glial form. The results provided here about the novel morphological changes concerning this specific astroglia in prion diseases are in agreement with those described for Alzheimer’s disease. The variations shown in bipolar disorder are less pronounced, and only messy or shortening of this cellular type was shown. Thus, this morphological alteration has been associated with neurological signs. Maybe the prodromal phase of prion diseases including neurological symptoms [67] could be reflecting these astroglial changes, as pointed out by other authors [68]. 

The main pathological changes that have been characterized in this peculiar astroglia include palisade disorganization, even breakdown, and the dramatic presence of thicker terminal masses. In those cases where the breakdown of the characteristic palisade of this astroglial type is evidenced, this neuropathological change might be triggered by alteration of the homeostasis associated with prion protein presence. Disruption, or even absence, described in this peculiar astroglial type, might also concur with subsequent images of a continuous process. The presence of thicker terminal masses observed in some CJD cases here had been detected in aged or adult [63] as well as in Alzheimer’s disease affected cases [64]. Despite the physiological changes occurring in these cells still remain unknown, its explanation seems to consist of constituting a structure where a re-emplacement of intermediate filaments occurs. Similarly, varicose astroglia observed in cerebella show a morphology that could resemble NG2 cells. NG2 represents an oligodendroglial progenitor marker, even considered to be the fourth glial type with astroglia, microglia, and oligodendroglia [69]. The hypothesis that astrocytes alter their morphology until they are replaced by these progenitor cells in an attempt to repair brain damage by compensating neuronal loss should be investigated. 

As cited, microglia show two pathological responses: (1) Graded morphological changes representing an increase in their physiological activity as a consequence of the neurodegenerative process and (2) morphological alterations, such as dystrophic or rod-like, of specific microglial populations that maybe show cellular injury as tisular homeostasis due to an indirect effect of the neurodegenerative process. In the same way as microglia, two alternative justifications could be proposed for astrocytic alterations: (1) Hypertrophy and/or hyperplasia of astrocytes as response to damage, defined as astrogliosis and (2) affectation of tissue homeostasis in specific astrocytic population, as interlaminar glia, caused by brain damage.

## 4. Materials and Methods

### 4.1. Samples

All tissue samples were kindly provided by the MRC Edinburgh Brain & Tissue bank (Edinburgh, UK - Brain Bank 16-ES-0084). Samples included 21 cases with a neuropathologically confirmed diagnosis of human prion disease and consisted of cases affected by different subtypes of CJD (sCJD, vCJD, gCJD, iCJD) and VPSPr. Where available, different PRNP codon 129 genotypes were included in the cases selected. Three additional non-dementia cases were included as controls (Table 2).

Formalin-fixed tissue samples of 3–5 mm, corresponding to sagittal cerebellar (including granular, Purkinje cell and molecular layers, and white matter) and frontal cortex (including I to VI layers and white matter) samples, were investigated in the study. In Biosafety Level-3 (BSL3) facilities, tissue samples were treated by immersion in formic acid for 1 h for prion inactivation before processing to paraffin embedded-tissue blocks.

Five micrometer tissue sections from all samples were sectioned for hematoxylin and eosin staining (H/E) and immunostaining with several antibodies following previously described protocols [38].

Research protocols applied on these samples were approved by the Ethical Committee for Clinical Research from Government of Aragón (CEICA; REFERENCE NUMBER: PI 15/0036, Acta Nº 05/2015).

### 4.2. GFAP Immunolabelling

Briefly, tissue sections were deparaffinized and rehydrated prior to endogenous peroxidase inactivation by incubation of all sections with peroxidase blocking reagent (5 min; DAKO, Glostrup, Denmark). Sections were incubated with a primary antibody against glial fibrillary acidic protein (GFAP, 1/500 for 30 min RT; DAKO), followed by an incubation with enzyme-conjugated polymer EnvisionTM (30 min, RT; DAKO). DAB PLUS (10 min) was used to visualize staining before sections were counter-stained with hematoxylin.

### 4.3. Reactive Microglia Detection

The protocol used for reactive microglia labelling was as that described for GFAP, with the exception of primary antibody. In this case, CD68 (1/500, 30 min RT; DAKO) and MHCII (1/200, 30 min RT; DAKO) were used.

### 4.4. PrP^sc^ Detection

PrP^sc^ immunodetection requires an epitope unmasking protocol consisting of immersion in 96% formic acid (5 min), digestion with proteinase K (5 min, RT; Roche, Reinach, Switzerland) and autoclaving in distilled water at 121 °C (10 min). After incubation with the prion protein antibody (12F10 at 1/1700 dilution; SPI Bio, Darmstadt, Germany) for 1 h RT, immunostaining was completed using the EnVision™ mouse polymer (DAKO, Glostrup, Denmark) kit. Following the previously described scoring system [38], all immunolabelled sections were thoroughly assessed in each layer regarding morphology, intensity, and distribution variations.

A semi quantitative scoring system was used in accordance with that described in Monzón et al. [38], in which scores were from negative (−) to a maximum (++++) according to the density and the extension of the labeling deposits. Spongiosis was also subjectively scored according to the number of vacuoles, from absence (−) to very high density (++++). Furthermore, immunostaining morphology for each marker was classified in accordance with the previously described patterns. For PrP^sc^ this included: Linear, granular, spot, coalescent or plaques, pericellular, patchy, perivacuolar, perivascular, plaque-like, plaque (kuru, unicentric, multicentric, florid plaque, microplaques), coarse, or synaptic; for GFAP: Severe diffuse, moderate, perineuronal, or radial [20] and for reactive microglia: Ramified, amoeboid, dystrophic, or rod-like. 

### 4.5. Statistical Analysis

Rating data were entered into an Excel spreadsheet and analyzed by *Servicio de Apoyo Metodológico y Estadístic*o (SAME)–IACS, University of Zaragoza. 

With the aim of testing for normality of the distribution, the Kolmogorov Smirnov test was conducted. Kruskal–Wallis test was performed for analysis. As significant differences between the groups were detected, the Mann Whitney U test post hoc testing (Bonferroni correction) was performed to identify the groups between which significant differences existed.

The correlation between all the variables analyzed was measured by Pearson’s correlation. 

*P* values < 0.05 were considered statistically significant.

### 4.6. Electron Microscopy Studies

With the aim of visualizing potential morphological alterations between the different CJD types, an ultrastructural study was performed. Post-fixation with glutaraldehyde 2.5% and 2% osmium before embedding in araldite (Durcupan, Fluka AG, Buchs SG, Switzerland) was followed in all cases. Ultrathin (0.08 μm) sections were cut from 1% toluidine blue-stained semi-thin sections (1.5 μm), collected on Formvar-coated single-slot grids and counterstained with 1% uranyl acetate and Reynold’s lead citrate for 10 min. Samples were examined under FEI Tecnai G2 Spirit (60,000 KV). 

## 5. Conclusions

In conclusion, the results provided here point to the fact that astroglial and microglial intensity and extension are independent of their morphological changes. Consequently, the relevance of studies about morphological changes, usually undervalued, should be confirmed as indisputable in order to understand the neuropathological events related to glial behavior. The cellular phenotype truly reveals specific cellular function. 

Although most authors presenting morphological alterations have intended to use their differences in order to classify and sub-classify specific neurodegenerative disease, we believe that focusing on common features contributes to our understanding of neuropathological process based on the newly emerging neuroinflammatory hypothesis. 

The increase of both glial cell types (astroglia/microglia) and the relationship with the increase in histopathological lesions suggest a potential neurotoxicity of glial cells in prion diseases, as described by Liddlelow et al. [27]. To determine whether astrocytes driven by ‘activated’ microglia or astrocyte dysfunction are the actual responsible actors for this neurotoxicity, urgent study is required. 

## Figures and Tables

**Table 1:** Correlation among spongiosis, PrP^sc^ accumulation, astrogliosis, and microgliosis in all CJD types analyzed in the study (Pearson’s correlation).

		PrP^sc^	GFAP	Microglia
Spongiosis	Pearson’s correlation	0.301 **	0.164 **	0.205 **
	*p*-value	0.000	0.008	0.001
PrP^sc^	Pearson’s correlation	1.00	−0.06	0.09
	*p*-value		0.313	0.127
GFAP	Pearson’s correlation		1.00	0.219 **
	*p*-value			0.000

** *p* < 0.01

**Table 2:** Data provided by respective Brain Bank corresponding to patients affected by each CJD type.

Patient	Aetiology	Gender	Age	Genetic Data	PrP Isotype
1	Sporadic	M	70	MM nil	2
2	Sporadic	M	70	MM nil	2
3	Sporadic	F	78	MM nil	1
4	Sporadic	M	71	MV nil	2
5	Sporadic	F	63	MV nil	2
6	Sporadic	F	71	MV nil	2
7	Sporadic	M	74	VV nil	2
8	Sporadic	F	73	VV nil	2
9	Sporadic	F	77	VV nil	2
10	Familial	F	56	MM E200K	na
11	Familial	M	78	MV E200K	na
12	Familial	F	53	MM E200K	na
13	Iatrogenic (GHT)	M	42	MM nil	1
14	Iatrogenic (GHT)	F	34	MV nil	1 + 2
15	Iatrogenic (DURA)	F	27	MM nil	1
16	Variant	M	59	MM nil	2B
17	Variant	M	62	MM nil	2B
18	Variant	F	32	MM nil	2B
19	VPSPr	M	66	VV	
20	VPSPr	F	76	VV lMWt	
21	VPSPr	M	66	MV	
22	Control	F	69		
23	Control	M	63		
24	Control	M	74		

PrP, prion protein; na, not available data; MM, MV, VV (M, methionine; V, valine): genotypes at codon 129 of PrP gene; Mutations are indicated when PrP gene mutation present; Nil, when no present.
